# Association between geniquin therapy and the risk of developing periodontal disease in patients with primary Sjögren’s syndrome: A population-based cohort study from Taiwan

**DOI:** 10.1371/journal.pone.0305130

**Published:** 2024-08-07

**Authors:** Chun-Yuan Chiu, Da-Yo Yuh, Li-Chyun Yeh, Iau-Jin Lin, Chi-Hsiang Chung, Chung-Hsing Li, Wu-Chien Chien, Gunng-Shinng Chen

**Affiliations:** 1 Department of Orthodontics and Pediatrics Dentistry, Tri-Service General Hospital, Taipei, Taiwan; 2 School of Dentistry, National Defense Medical Center, Taipei, Taiwan; 3 Department of Periodontology, Tri-Service General Hospital, Taipei, Taiwan; 4 School of Early Childhood Care and Education, University of Kang-Ning, Taipei, Taiwan; 5 Graduate Institute of Life Sciences, National Defense Medical Center, Taipei, Taiwan; 6 School of Public Health, National Defense Medical Center, Taipei, Taiwan; 7 Taiwanese Injury Prevention and Safety Promotion Association, Taipei, Taiwan; 8 Department of Medical Research, Tri-Service General Hospital, National Defense Medical Center, Taipei, Taiwan; University of Pennsylvania, UNITED STATES

## Abstract

Primary Sjögren’s syndrome (pSS) is a chronic autoimmune disease that causes dysfunction of salivation and harmful oral conditions. The association between periodontal disease (PD) and pSS with or without geniquin therapy remains controversial. This study evaluated the association between geniquin therapy and the risk of subsequent development of PD in pSS patients. From Taiwan’s National Health Insurance Research Database, we selected a control cohort of 106,818 pSS patients, followed up from 2000 to 2015, matched (1:4) by age and index year with 427,272 non-pSS patients. We also analyzed 15,149 pSS patients receiving geniquin therapy (cohort 1) and 91,669 pSS patients not receiving geniquin therapy (cohort 2). After adjusting for confounding factors, multivariate Cox proportional hazards regression analysis was used to compare the risk of PD over the 15-year follow-up. In the control cohort, 11,941 (11.2%) pSS patients developed PD compared to 39,797 (9.3%) non-pSS patients. In cohorts 1 and 2, 1,914 (12.6%) pSS patients receiving geniquin therapy and 10,027 (10.9%) pSS patients not receiving geniquin therapy developed PD. The adjusted hazard ratio (HR) for subsequent PD in pSS patients was 1.165 (95% confidence interval [CI] = 1.147–1.195, *p* < 0.001) and in pSS patients receiving geniquin therapy was 1.608 (95% CI = 1.526–1.702, *p* < 0.001). The adjusted HR for PD treatment was 1.843. Patients diagnosed with pSS showed an increased risk of developing subsequent PD and receiving PD treatment than patients without pSS, while pSS patients receiving geniquin therapy showed even higher risks.

## Introduction

Primary Sjögren’s syndrome (pSS) is a systemic autoimmune disorder affecting between 0.9 to 6.0 per 1000 individuals [1]. It is characterized by lymphoid infiltration of exocrine glands that reduces lacrimal and salivary fluxes. It predominantly affects females, with a female-to-male ratio of 9:1, and incidence peaks at approximately 50 years of ag [2]. An increasing number of studies have shown that several pathophysiological changes occur in the salivary flow rate and salivary composition [3–5]. Decreased salivary gland function can lead to oral dryness (xerostomia), which is a major dental clinical characteristic of patients with pSS [6]. Symptoms of xerostomia—dysphagia, lack of taste, and speech problems—in patients with pSS can lead to a poor quality of life and proneness to developing progressive dental decay and inflammation of the oral mucosa [7].

Periodontal disease (PD) is a chronic inflammatory condition characterized by the destruction of periodontal tissue, which leads to the loss of connective tissue attachment and alveolar bone and the formation of pathological pockets around the diseased teeth. It is common in adults aged 50 years or older [8]. Changes in the severity of PD have been reported for patients with various autoimmune diseases, including systemic lupus erythematosus (SLE) [9], rheumatoid arthritis (RA) [10], and pSS [11, 12]. The hyperactive immune responses of autoimmune diseases may lead to deterioration of the periodontal condition.

Geniquin (hydroxychloroquine) is a derivative of quinine, an extract of the cinchona tree, which was first used to treat malaria and is now commonly used as a therapy for autoimmune diseases. It is the most frequently prescribed immunomodulatory drug for primary Sjögren’s syndrome [13] as a treatment for fatigue, arthralgia, and myalgia [14], rather than for severe systemic manifestations. Hydroxychloroquine has also been shown to have an anti-inflammatory effect on other autoimmune diseases such as SLE and RA [15, 16], but there are no relevant studies involving patients with periodontitis.

Previous population studies have shown an association between pSS and periodontitis [17, 18]. However, these studies did not demonstrate an effect of pSS drugs on periodontitis. This study analyzed the association of periodontitis with geniquin therapy for pSS. It compared patients with pSS to those without pSS and pSS patients receiving geniquin therapy and those that did not to patients without pSS over a 15-year follow-up period.

## Materials and methods

### Data sources

This retrospective population-based controlled study used insurance claim data obtained from the Longitudinal Health Insurance Database of Taiwan. The National Health Insurance (NHI) Program was launched in Taiwan in 1995, and as of June 2009, it provided cover for approximately 23 million beneficiaries, or more than 99% of the population of Taiwan, and had contracts with 97% of the medical providers in the country [19]. The Taiwan National Health Insurance Research Database (NHIRD) provides data for 2 million randomly sampled beneficiaries. The legitimacy of the data from NHIRD is supported by published studies [20–24]. Diseases (comorbidities and outcomes) were defined in this study according to the International Classification of Diseases, Ninth Revision, Clinical Modification (ICD-9-CM) [25]. Although smoking status, alcohol use, and laboratory and radiographic information are not available in this database, the NHIRD routinely assesses the accuracy of diagnoses by randomly sampling patient charts to enhance coding accuracy [26]. This study was fully reviewed and approved by the Tri-Service General Hospital Joint Institutional Review Board (No. E202216014). The NHIRD data were accessed for research purposes in June 24, 2022, and the authors had no access to information that could identify individual participants during or after data collection. Participant consent was waived due to the retrospective nature of this study.

### Study sample

Patients diagnosed with pSS (ICD-9-CM code: 710.2) between January 1, 2000, and December 31, 2015, were assigned the first index date. pSS was diagnosed according to the criteria developed by the American–European Consensus Group (2002); the Group postulated six criteria, based on oral and ocular symptoms and signs, the histopathology of salivary glands, glandular dysfunction, and the presence of autoantibodies anti-Ro (SSA) and anti-La (SSB). At least four of the six criteria must be positive for a diagnosis of pSS [27], but not according to the new classification proposed in 2012 by the Sjögren’s International Collaborative Clinical Alliance cohort [28]. We also excluded secondary Sjögren’s syndrome in the presence of RA (ICD-9-CM code: 714.0), SLE (ICD-9-CM code:710.0), sarcoidosis (ICD-9-CM code:135), polymyositis (ICD-9-CM code: 710.4), dermatomyositis (ICD-9-CM code: 714.3), and systemic sclerosis (ICD-9-CM code: 710.1).

**Fig 1:**
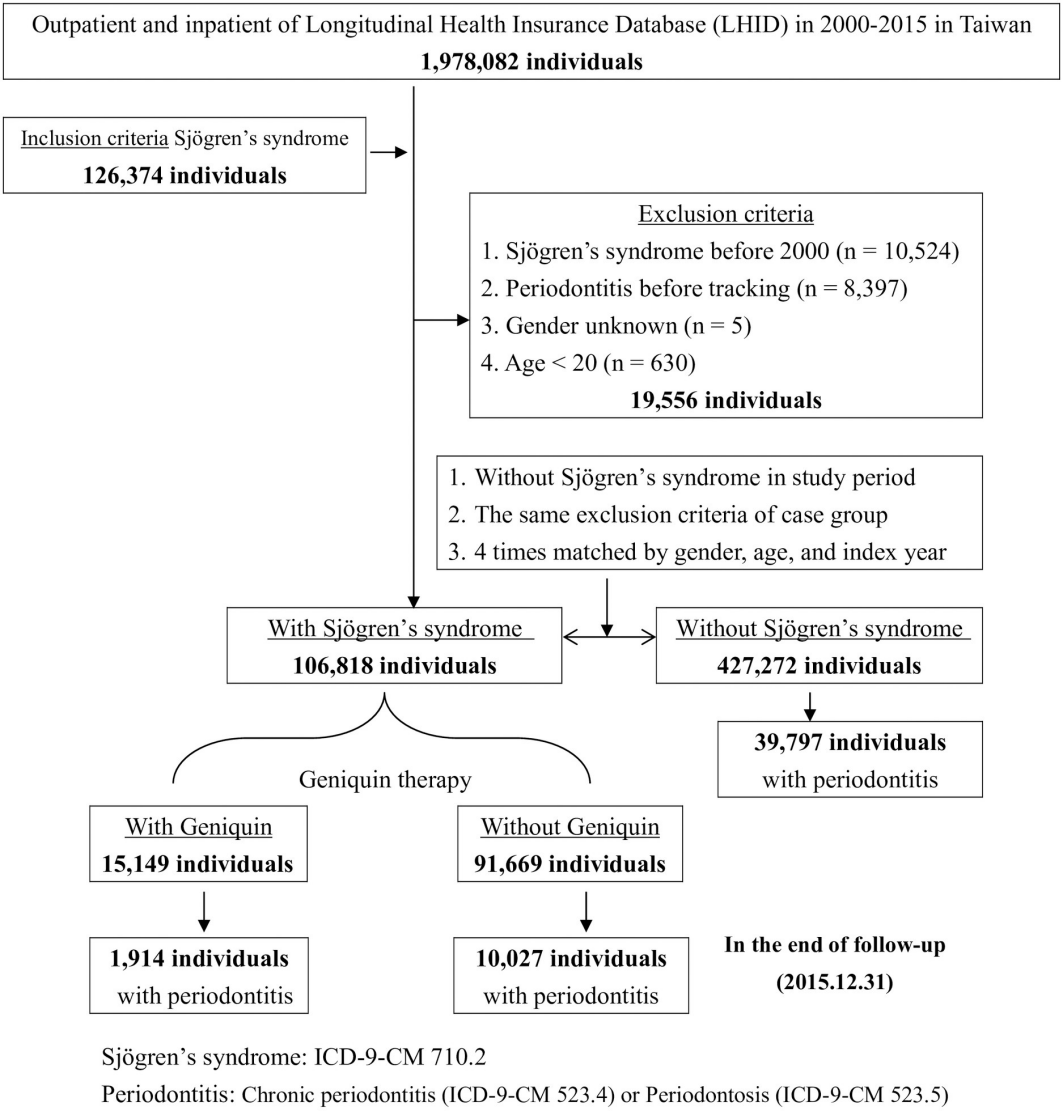
The flowchart of study sample selection from the National Health Insurance Research Database in Taiwan.

Of the 126,374 pSS patients, 19,556 individuals fitting the following criteria were excluded: SS history before 2000 (*n* = 10,524), periodontitis before tracking (*n* = 8,397), unknown sex (*n* = 5), and aged <20 years (*n* = 630). Because its distribution of patient characteristics differed considerably from the compared cohort group, we matched the non-pSS comparison cohort by sex, age, and index year based on a 1:4 ratio. The total number of patients with pSS was 106,818. Of these patients, 11,941 (11.2%) had PD and 15,149 were treated with geniquin therapy; 1,914 (12.6%) of the patients treated with geniquin were with PD. Of the 91,669 pSS patients who did not receive geniquin therapy, 10,027 (10.9%) developed PD (Fig 1).

Patient characteristics included sociodemographic factors (gender, age, income, geographic region, and urbanization level), comorbidities (diabetes mellitus [DM], hypertension, depression, stroke, dementia, chronic kidney disease [CKD], osteoporosis, and heart disease), medication (cevimeline and pilocarpine), and revised Charlson comorbidity index (CCI-R) score [29]. Comorbidities were defined as either a diagnosis two or three times in the outpatient department or submission of an outpatient claim for the same individuals up to one year before the index date.

### Identification of patients with periodontal disease

In Taiwan, patients with a concurrent coding of PD (ICD9-CM Codes 523.4–5) can receive a regular dental examination and full-mouth scaling twice per year. In this study, we excluded patients with a history of PD (ICD9-CM Codes 523.4–5) before the index date who had a diagnosis of pSS. Each patient with PD in the study was required to have made at least three outpatient visits with the 523.4–5 code being filed at least three times within the first year. These patients received NHI order codes 91006–91008C (subgingival curettage/root planing) or 91009B–91010B (periodontal flap operation) and were defined as the PD therapy subgroup [30]. Children and adolescents with PD are less common, and we also excluded patients under 20 years of age.

### Statistical analysis

**Fig 2:**
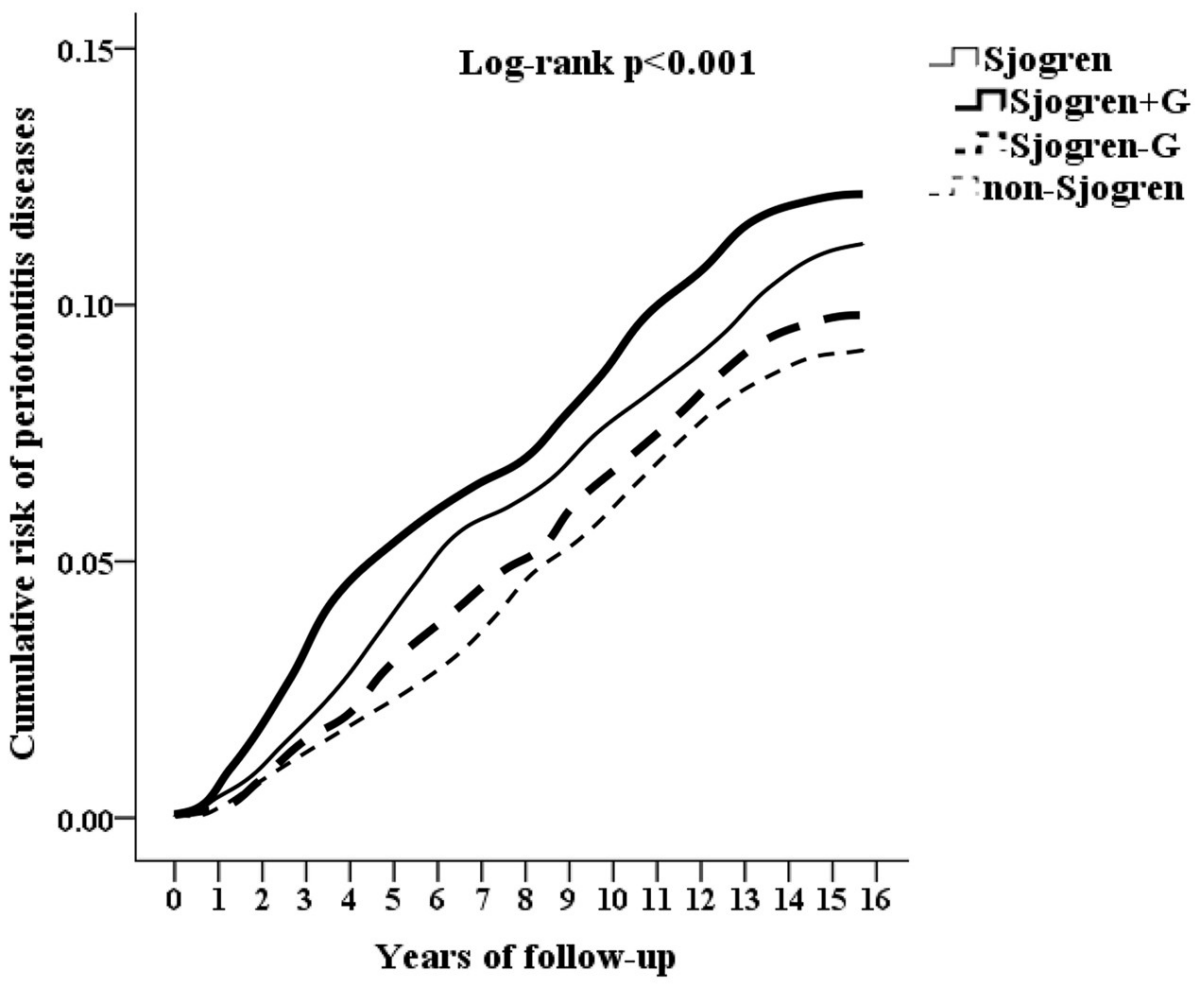
Kaplan–Meier curves for cumulative risk of periodontal disease in patients aged ≥ 20 years stratified by study group and compared using the log-rank test.

The Kaplan–Meier hazard model and log-rank test were used to examine the association between pSS and PD after adjustment of covariates (Fig 2). A two-tailed *p*-value of <0.05 was considered statistically significant. All statistical analyses were performed using SPSS software version 22 (SPSS Inc., Chicago, Illinois, USA).

## Results

### Characteristics of the study groups at baseline

The majority of the 73,391 (68.7%) patients with pSS in the control cohort were female, of middle age, living in northern Taiwan (48.7%) in urbanization level 2 areas (31.3%), and receiving medical care in clinic (56.9%) (Table 1). The proportion of pSS patients with a low income (0.8 vs 1.0%), DM (20.9 vs 26.4%), hypertension (35.1 vs 37.1%), stroke (1.6 vs 2.3%), dementia (3.3 vs 4.1%), or CKD (12.3 vs 14.2%) was lower and the proportion with depression (12.6 vs 9.5%), heart disease (26.0% vs 21.9%), cevimeline (15.7% vs 8.8%), or pilocarpine (16.5% vs 9.7%) was higher than that of the control group.

**Table 1:** Characteristics of the study groups at baseline.

Variables/Group	Control Cohort—Sjögren’s vs. Non-Sjögren’s			Cohort 1	Cohort 2
	Total	Sjögren’s	Non-Sjögren’s	Sjögren’s w/ Geniquin	Sjögren’s w/o Geniquin
	(N = 534,090)	(N = 106,818)	(N = 427,272)	(N = 15,149)	(N = 91,669)
**Gender Female**	68.7%	68.7%	68.7%	78.7%	67.1%
**Male**	31.3%	31.3%	31.3%	21.3%	32.9%
**Age** (years)	50.92±16.19	50.92±16.19	50.92±16.19	51.58±14.98	50.81±16.38
**CCI-R**	1.88±2.18	1.88±2.18	1.88±2.18	2.33±2.13	1.8±2.18
**Age** 18–39	26.5%	26.5%	26.5%	22.0%	27.2%
40–64	51.3%	51.3%	51.3%	57.6%	50.3%
≧65	22.2%	22.2%	22.2%	20.4%	22.5%
**Low-income** (With)	0.9%	0.8%**	1.0%	0.6%**	0.8%**
**DM**(With)	25.3%	20.9%**	26.4%	20.4%**	21.0% **
**HTN**	36.7%	35.1%**	37.1%	35.0% **	35.1% **
**Depression**	10.1%	12.6%**	9.5%	14.7%**	12.2%**
**Stroke**	2.2%	1.6%**	2.3%	1.5%**	1.6%**
**Dementia**	3.9%	3.3%**	4.1%	3.6%**	3.3%**
**CKD**	13.8%	12.3%**	14.2%	12.8% **	12.2% **
**Osteoporosis**	11.0%	13.2%	10.5%	12.1%	13.4%
**Heart disease**	22.7%	26.0%*	21.9%	29.7%*	25.4%*
**Cevimeline**	10.2%	15.7%**	8.8%	17.8%**	15.4%**
**Pilocarpine**	11.1%	16.5%**	9.7%	18.1%**	16.2%**
**Location**					
**Northern** (Taiwan)	49.2%	48.7%	49.3%	44.8%	49.3%
**Middle**	18.6%	25.0%	17.0%	26.1%	24.8%
**Southern**	29.4%	23.3%	31.0%	24.4%	23.1%
**Eastern**	2.4%	2.7%	2.4%	4.4%	2.4%
**Outlets islands**	0.4%	0.3%	0.4%	0.3%	0.3%
**Urbanization level**					
**1** (Highest)	28.6%	29.8%	28.3%	27.4%	30.1%
**2**	31.6%	31.3%	31.6%	32.6%	31.1%
**3**	29.9%	30.0%	29.9%	29.8%	30.0%
**4** (Lowest)	9.9%	9.0%	10.2%	10.2%	8.8%
**Level of care**					
Hospital Center	13.8%	15.6%	13.4%	37.1%	12.0%
Regional Hospital	16.1%	14.6%	16.5%	26.3%	12.6%
Local Hospital	13.6%	12.9%	13.8%	16.2%	12.4%
Clinic	56.4%	56.9%	56.3%	20.4%	63.0%

P: Chi-square/Fisher exact test on category variables and t-test on continue variables.

*The pair of subsets showed a significant difference at p<0.05.

** The pair of subsets showed a significant difference at p<0.001.

DM: diabetes mellitus, HTN: hypertension, CKD: chronic kidney disease

In cohort 1 (patients with pSS receiving geniquin therapy), the proportion of pSS patients with a low income (0.6%) was lower and that with depression (14.7%), heart disease (29.7%), cevimeline (17.8%), or pilocarpine (18.1%) was higher compared to control-group patients, and more patients received medical care at a hospital center (37.1%). In contrast, cohort 2 (pSS patients not receiving geniquin therapy) was similar to the control cohort in most of these parameters (Table 1).

### Characteristics of the study groups at the end of follow-up

In the control group, higher proportions of patients with pSS had PD and received periodontal therapy than patients without pSS (11.2 vs 9.3% and 5.6% vs 4.6%, respectively) at the end of the follow-up period (Table 2). Patients with pSS also had higher CCI-R scores (2.71 ± 2.65 vs 2.47 ± 2.54), a higher rate of depression (12.6 vs 9.5%), heart disease (26.0% vs 21.9%), cevimeline (15.7% vs 8.8%), and pilocarpine (16.5% vs 9.7%) and lower rates of DM (20.9 vs 26.4%), hypertension (35.1 vs 37.1%), stroke (1.6 vs 2.3%), dementia (3.3 vs 4.1%), and CKD (12.3 vs 14.2%) compared to patients without pSS in the control group.

**Table 2:** Characteristics of the study groups at the end of follow-up.

Variables/Group	Control Cohort—Sjögren’s vs. Non-Sjögren’s			Cohort 1	Cohort 2
	Total	Sjögren’s	Non-Sjögren’s	Sjögren’s w/ Geniquin	Sjögren’s w/o Geniquin
	(N = 534,090)	(N = 106,818)	(N = 427,272)	(N = 15,149)	(N = 91,669)
**Periodontitis**	9.7%	11.2%**	9.3%	12.6%**	10.9%**
**Periodontal therapy**	4.8%	5.6%**	4.6%	6.4%**	5.5%**
**Gender Female**	68.7%	68.7%	68.7%	78.7%	67.1%
**Male**	31.3%	31.3%	31.3%	21.3%	32.9%
**Age** (years)	57.81±16	58.01±16.13*	57.76±15.96	58.66±14.85	57.91±16.34*
**CCI**	2.52±2.56	2.71±2.65**	2.47±2.54	3.21±2.57**	2.62±2.65**
**Age** 18–39	16.2%	16.1%	16.2%	12.4%	16.7%
40–64	50.7%	50.2%	50.8%	54.3%	49.5%
≧65	33.2%	33.7%	33.1%	33.2%	33.8%
**Low-income** (With)	0.9%	0.8%**	1.0%	0.6%**	0.8%**
**DM**(With)	25.3%	20.9%**	26.4%	20.4% **	21.0% **
**HTN**	36.7%	35.1%**	37.1%	35.0% **	35.1% **
**Depression**	10.1%	12.6%**	9.5%	14.7%**	12.2%**
**Stroke**	2.2%	1.6%**	2.3%	1.5%**	1.6%**
**Dementia**	3.9%	3.3%**	4.1%	3.6%**	3.3%**
**CKD**	13.8%	12.3%**	14.2%	12.8% **	12.2% **
**Osteoporosis**	11.0%	13.2%	10.5%	12.1%	13.4%
**Heart disease**	22.7%	26.0%*	21.9%	29.7%*	25.4%*
**Cevimeline**	10.2%	15.7%**	8.8%	17.8%**	15.4%**
**Pilocarpine**	11.1%	16.5%**	9.7%	18.1%**	16.2%**
**Location**					
**Northern** (Taiwan)	49.2%	48.7%**	49.3%	44.8%	49.3%**
**Middle**	18.6%	25.0%	17.0%	26.1%	24.8%
**Southern**	29.4%	23.3%	31.0%	24.4%	23.1%
**Eastern**	2.4%	2.7%	2.4%	4.4%	2.4%
**Outlets islands**	0.4%	0.3%	0.4%	0.3%	0.3%
**Urbanization level**					
**1** (Highest)	28.6%	29.8%**	28.3%	27.4%	30.1%**
**2**	31.6%	31.3%	31.6%	32.6%	31.1%
**3**	29.9%	30.0%	29.9%	29.8%	30.0%
**4** (Lowest)	9.9%	9.0%	10.2%	10.2%	8.8%
**Level of care**					
Hospital Center	13.0%	14.5%	12.7%	31.8%	11.7%
Regional Hospital	14.8%	13.3%	15.2%	23.2%	11.7%
Local Hospital	12.2%	11.4%	12.4%	13.8%	11.0%
Clinic	60.0%	60.8%*	59.8%	31.2%**	65.7%**

P: Chi-square/Fisher exact test on category variables and t-test on continue variables.

*The pair of subsets showed a significant difference at p<0.05.

** The pair of subsets showed a significant difference at p<0.001.

DM: diabetes mellitus, HTN: hypertension, CKD: chronic kidney disease

Patients in cohort 1 (pSS patients treated with geniquin) had a higher rate of PD (12.6%) and periodontal therapy (6.4%), a higher CCI-R score (3.21 ± 2.57), higher rate of depression (14.7%), heart disease (29.7%), cevimeline (17.8%), or pilocarpine (18.1%), and were more likely to receive medical care at a hospital center (31.8%) than the control group. Patients in cohort 2 (patients with pSS not treated with geniquin) had a higher rate of PD (10.9%) and periodontal therapy (5.5%) and a higher proportion received medical care at a clinic (65.7%) than the control group.

### Risk factors of developing subsequent periodontal disease

Table 3 shows the results of Cox proportional hazards regression analysis of the factors associated with the risk of developing PD. The crude hazard ratio (HR) of the control cohort was 1.131 (95% confidence interval [CI] = 1.108–1.154, *p* < 0.001). After adjusting for gender, age, comorbidities, geographical area of residence, urbanization level of area of residence, and low income, the adjusted HR was 1.165 (95% CI = 1.141–1.194, *p* < 0.001).

**Table 3:** Cox proportional hazards regression analysis of risk factors of periodontal disease.

Variables/Group	Control Cohort pSS vs. Non-pSS		Cohort 1 pSS with Geniquin therapy vs. Non-pSS		Cohort 2 pSS without Geniquin therapy vs. Non-pSS	
	Crude HR	Adjusted HR	Crude HR	Adjusted HR	Crude HR	Adjusted HR
Sjögren‘s (vs Non-Sjögren‘s)	1.131(1.108–1.154) **	1.165 (1.141–1.194)**	1.239(1.177–1.305)**	1.608 (1.523–1.698)**	1.112(1.088–1.137) **	1.120 (1.098–1.149)**
Male(vs Female)	1.143(1.122–1.164) **	1.165 (1.133–1.182)**	1.235(1.171–1.303)**	1.263 (1.194–1.335)**	1.137(1.115–1.159) **	1.168 (1.135–1.187)**
Age (years)	1.003(1.003–1.004) **	0.998 (0.997–0.998)**	1.004(1.003–1.006)**	0.998 (0.995–0.999)*	1.003(1.002–1.004) **	0.997 (0.997–0.998)**
Post CCI-R	0.999(0.996–1.003)	0.954 (0.951–0.959)**	1.005(0.996–1.014)	0.950 (0.938–0.961)**	0.998(0.994–1.002)	0.952 (0.944–0.956)**
Low-income (vs Without)	1.109(1.000–1.229)*	1.166 (1.035–1.298)*	1.102(0.873–1.451)	1.243 (0.942–1.643)	1.110(0.994–1.240)	1.168 (1.033–1.301)*
DM (vs Without)	1.477(1.448–1.507)**	1.462 (1.427–1.491)**	1.507(1.433–1.585)**	1.482 (1.398–1.561)**	1.471(1.439–1.503)**	1.452 (1.420–1.496)**
HTN (vs Without)	1.422(1.397–1.448)**	1.388 (1.351–1.415)**	1.449(1.383–1.518)**	1.386 (1.309–1.464)**	1.417(1.389–1.445)**	1.388 (1.352–1.411)**
Depression (vs Without)	1.641(1.595–1.689)**	1.608 (1.557–1.658)**	1.700(1.587–1.822)**	1.618 (1.502–1.730)**	1.628(1.577–1.680)**	1.600 (1.551–1.652)**
Stroke (vs Without)	1.411(1.290–1.542)**	1.047 (0.953–1.151)	1.741(1.402–2.163)**	1.327 (1.062–1.633)*	1.357(1.230–1.497)**	1.013 (0.917–1.118)
Dementia (vs Without)	1.476(1.402–1.553)**	1.387 (1.314–1.460)**	1.416(1.250–1.604)**	1.358 (1.194–1.545)**	1.487(1.406–1.573)**	1.382 (1.306–1.471)**
CKD (vs Without)	1.382(1.345–1.420)**	1.265 (1.231–1.308)**	1.407(1.312–1.508)**	1.275 (1.180–1.372)**	1.377(1.337–1.418)**	1.273 (1.221–1.305)**
Osteoporosis (vs without)	1.098 (0.864–1.275)	1.050 (0.833–1.186)	1.057 (0.832–1.233)	1.022 (0.827–1.179)	1.104 (0.879–1.288)	1.059 (0.842–1.197)
Heart disease (vs without)	1.347 (1.264–1.578)**	1.325 (1.211–1.536)**	1.325 (1.243–1.562)**	1.318 (1.207–1.522)**	1.367 (1.280–1.594)**	1.329 (1.218–1.539)**
Cevimeline (vs without)	0.862 (0.521–1.411)	0.897 (0.534–1.424)	0.855 (0.510–1.396)	0.886 (0.528–1.411)	0.872 (0.530–1.424)	0.902 (0.549–1.433)
Pilocarpine (vs without)	0.895 (0.533–1.452)	0.913 (0.562–1.518)	0.887 (0.526–1.438)	0.908 (0.535–1.502)	0.909 (0.542–1.478)	0.925 (0.572–1.530)
Location (vs Northern Taiwan)						
Middle Taiwan	1.016(0.993–1.040)	Had collinearity with urbanization level	0.966(0.910–1.025)	Had collinearity with urbanization level	1.025(0.999–1.052)	Had collinearity with urbanization level
Southern Taiwan	0.916(0.897–0.935)**		0.885(0.840–0.933)**		0.921(0.901–0.941)**	
Eastern Taiwan	1.016(0.961–1.075)		0.905(0.787–1.041)		1.038(0.976–1.104)	
Urbanization level (vs level 1,highest)						
2	0.904(0.886–0.924) **	0.910 (0.891–0.928)**	0.880(0.834–0.930)**	0.864 (0.818–0.911)**	0.909(0.888–0.930)**	0.919 (0.892–0.938)**
3	0.771(0.754–0.789)**	0.768 (0.752–0.784)**	0.748(0.706–0.793)**	0.728 (0.685–0.773)**	0.775(0.757–0.794)**	0.771 (0.750–0.792)**
4 (Lowest)	0.654(0.631–0.677)**	0.631 (0.608–0.652)**	0.618(0.563–0.678)**	0.573 (0.521–0.630)**	0.660(0.635–0.686)**	0.638 (0.613–0.664)**
Level of care (vs Clinic)						
Hospital Center	0.661(0.643–0.678)**	0.613 (0.592–0.628)**	0.504(0.472–0.538)**	0.430 (0.402–0.463)**	0.695(0.675–0.716)**	0.642 (0.623–0.662)**
Regional Hospital	0.451(0.438–0.465)**	0.420 (0.408–0.435)**	0.362(0.335–0.392)**	0.325 (0.301–0.356)**	0.468(0.453–0.484)**	0.440 (0.425–0.454)**
Local Hospital	0.297(0.286–0.309)**	0.288 (0.274–0.300)**	0.266(0.240–0.294)**	0.251 (0.224–0.283)**	0.302(0.290–0.316)**	0.291 (0.281–0.307)**

HR = hazard ratio, CI = confidence interval, Adjusted HR: Adjusted variables listed in the table.

*The pair of subsets showed a significant difference at p<0.05.

** The pair of subsets showed a significant difference at p<0.001.

DM: diabetes mellitus, HTN: hypertension, CKD: chronic kidney disease

### Increased risk of periodontal disease of pSS patients, higher risk of periodontal disease associated with geniquin therapy

Patients in cohort 1 (patients with pSS receiving geniquin therapy) had a 1.612 times higher risk of PD (95% CI = 1.526–1.702, *p* < 0.001) than patients without pSS in the control group. The adjusted HRs of male patients and patients with a low income, DM, hypertension, depression, stroke, dementia, CKD, and heart disease were 1.275 (*p* < 0.001), 1.251 (*p* < 0.001), 1.483 (*p* < 0.001), 1.390 (*p* < 0.001), 1.625 (*p* < 0.001), 1.336 (*p* < 0.001), 1.365 (*p* < 0.001), 1.280 (*p* < 0.001), and 1.318 (*p* < 0.001), respectively. pSS patients receiving treatment at hospital centers (0.434, *p* < 0.001), regional hospitals (0.330, *p* < 0.001), or local hospitals (0.256, *p* <0.001) tended to have a lower risk of developing PD than those who visited a local clinic.

Patients in cohort 2 (patients with pSS not receiving geniquin therapy) had a 1.120 times higher risk of PD (95% CI = 1.098–1.149, *p* < 0.001) than the patients without pSS in the control group. Most of the adjusted HRs were similar to those of the control cohort.

### Risk factors of periodontal disease and periodontal therapy

Table 4 shows the results of Cox regression analysis of the risks of PD and periodontal therapy. The adjusted HRs for periodontal therapy of the control cohort (pSS vs non-pSS), cohort 1 (pSS with geniquin therapy), and cohort 2 (pSS without geniquin therapy) were 1.195 (95% CI = 1.157–1.227, *p* < 0.001), 1.843 (95% CI = 1.720–1.990, *p* < 0.001), and 1.138 (95% CI = 1.104–1.172, *p* < 0.001), respectively.

**Table 4:** Cox proportional hazards regression analysis of risk factors of periodontal disease at the end of follow-up.

**Subgroup**	**pSS**	**Non-pSS**	**pSS vs. Non-pSS**		
	n	n	**Adjusted HR**	**95% CI**	**P**
Periodontitis	11,941	39,797	1.165	1.141–1.194	<0.0001
Periodontal therapy	6,007	19,828	1.195	1.157–1.227	<0.0001
**Subgroup**	**pSS w/ Geniquin**	**Non-pSS**	**pSS with Geniquin therapy vs. Non-pSS**		
	n	n	**Adjusted HR**	**95% CI**	**P**
Periodontitis	1,914	5,882	1.608	1.523–1.698	<0.0001
Periodontal therapy	966	2,942	1.843	1.720–1.990	<0.0001
**Subgroup**	**pSS w/o Geniquin**	**Non-pSS**	**pSS without Geniquin therapy vs. Non-pSS**		
	n	n	**Adjusted HR**	**95% CI**	**P**
Periodontitis	10,027	33,915	1.120	1.098–1.149	<0.0001
Periodontal therapy	5,041	16,886	1.138	1.104–1.172	<0.0001

HR = hazard ratio, CI = confidence interval, Adjusted HR: Adjusted variables listed in Table 3.

S1 Table shows that PD (12.6% vs 10.9%, *p* < 0.001) and periodontal therapy (6.4% vs 5.5%, *p* < 0.001) were more common in pSS with than without geniquin therapy at the end of follow-up.

## Discussion

This study identified a significantly higher prevalence of PD in individuals diagnosed with primary Sjögren’s syndrome (pSS). Among pSS patients, the PD rate stood at 11.2%, representing a 1.165 times greater risk than the control group, where the PD rate was 9.3%. Remarkably, pSS patients undergoing geniquin therapy exhibited an even greater PD prevalence at 12.6% and a higher risk of 1.608 times after a 15-year follow-up. Notably, this study marks the first population-based examination of the increased PD risk associated with geniquin therapy in pSS patients.

A range of potential pathophysiological mechanisms could explain the elevated risk of PD in pSS patients. One likely factor is the hypofunction of salivary glands in pSS patients. Exocrinopathy in pSS results in hyposalivation, creating a favorable environment for opportunistic pathogens such as *Streptococcus mutans* and *Candida albicans* [31] to thrive. The absence of enzyme systems and self-cleansing processes typically found in saliva can lead to distinct clinical manifestations like PD [32, 33].

It’s noteworthy that no disparities have been observed in periodontal conditions concerning the presence of microorganisms in the gingival sulcus between patients with primary or secondary Sjögren’s syndrome and healthy individuals [33].

The connection between salivary flow and PD remains a topic of interest. Animal studies, such as those involving Syrian hamsters, have shown a significant increase in PD incidence following the removal of salivary glands [34]. However, human studies have not consistently established a direct relationship between major salivary gland flow rates and gingival/periodontal conditions [35]. Although there have been differences in the plaque index between patients with pSS and those with xerostomia, no variations in the gingival index, bleeding on probing index, or pocket depth have been found. Consequently, the precise link between salivary flow and PD development remains to be definitively established.

In conclusion, this study underscores the heightened risk of PD in pSS patients, particularly those undergoing geniquin therapy, and sheds light on the possible role of salivary gland hypofunction in this relationship [36]. Further research is needed to elucidate the intricate mechanisms connecting these two conditions.

The autonomic nervous system (ANS) serves as a vital mechanistic link in the development of PD in individuals with primary Sjögren’s syndrome (pSS), marking the second significant connection. In pSS, ANS dysfunction has been associated with various immunological factors [37–42]. Autoimmunity, among these factors, is believed to influence ANS function and enhance its involvement in pSS pathogenesis [27, 43].

The ANS is integral to regulating exocrine gland secretions, and disruptions in its signaling pathways, as proposed by Konttinen and colleagues in the early 1990s [44], may explain the incongruity between exocrine gland morphology and function in pSS. A physiological response in pSS can activate the ANS, leading to the release of catecholamines, such as epinephrine and norepinephrine, which stimulate the production and activity of prostaglandins and proteolytic enzymes, potentially leading to tissue damage [45].

Additionally, catecholamines can alter the function of immune cells, resulting in increased levels of pro-inflammatory cytokines like interleukin-1 (IL-1), IL-2, IL-3, IL-6, tumor necrosis factor alpha (TNF-α), and interferon gamma (IFN-γ) [46, 47], which may contribute to the development of PD [48]. This intricate interplay between ANS dysfunction and immunological factors represents a crucial link in understanding the elevated risk of PD in pSS patients.

A third potential mechanism linking primary Sjögren’s syndrome (pSS) and PD revolves around immune system-inflammatory regulation. One avenue of this connection lies in the diminished expression of developmental endothelial locus-1 (DEL-1) within salivary glands and periodontal tissue. DEL-1, a glycoprotein with a well-documented role in immune regulation and tissue homeostasis, has garnered attention for its involvement in various autoimmune conditions [49] and periodontal health [50]. Baban and colleagues demonstrated reduced DEL-1 expression in salivary glands in both a murine model and pSS patients, potentially suggesting a link between pSS and periodontitis [51].

The murine model further emphasized an increase in IL-12, a Th1 proinflammatory cytokine, concomitant with immune dysfunctions in pSS [52]. As a result, systemic immunologic alterations in pSS may influence immune responses to bacterial challenges within the periodontal environment. Notably, a previous study revealed that, after adjusting for PD, individuals with pSS exhibited lower levels of IL-1β compared to controls [53].

Another hypothesis implicating immune dysfunction in pSS in the context of periodontal challenges may be pertinent to the PD link. Clinical parameters in periodontitis have been correlated with elevated levels of the B-cell activating factor in saliva, suggesting its potential role in PD pathogenesis [36]. Patients with pSS also demonstrate significantly increased serum antibodies against *A*. *actinomycetemcomitans* and *P*. *gingivalis* compared to controls [54]. However, a separate investigation into antibody responses to six bacteria associated with periodontitis did not identify distinct antibody responses to PD-related bacteria in pSS, nor did it establish an elevated prevalence of PD in pSS patients [55].

The gender distribution among primary Sjögren’s syndrome (pSS) patients reveals a female-to-male ratio of approximately 2:1, whereas PD is more commonly observed in males. This observation can be partly attributed to the increased prevalence of smoking among males, a well-established risk factor for PD development [56, 57]. It is essential to acknowledge that this study is subject to the limitation of this confounding factor. To gain a more comprehensive understanding of the results and to assess the potential impact of various factors on periodontal status, it is imperative to account for possible risk factors. These include variables like dental plaque levels, diabetes mellitus, and smoking, which should be considered in the analysis [58].

Hydroxychloroquine(HCQ) is utilized for systemic Sjögren’s syndrome (SS) treatment, offering potential benefits by enhancing salivary gland function through cholinesterase activity inhibition and antigen processing interference [59–61]. A systematic review and meta-analysis support its efficacy, particularly in alleviating oral symptoms in primary Sjögren’s syndrome (pSS) [62].

While a prior study found increased unstimulated salivary flow rates in pSS patients with hydroxychloroquine treatment, stimulated salivary flow and subjective dryness complaints showed minimal changes [63]. Conversely, a recent randomized clinical trial did not reveal significant differences in ocular and oral dryness, as assessed by the Schirmer test, or unstimulated salivary flow in pSS patients undergoing hydroxychloroquine therapy compared to a placebo group [64].

A recent study demonstrated that HCQ suppressed bone mineral resorption in vitro and decreased the bone resorption marker β-CTx in vivo, hypothesizing that HCQ induces osteoclastic lysosomal membrane permeabilization, leading to decreased bone resorption [65]. Another population-based study found that HCQ use was associated with a reduced risk of new-onset DM in patients with Sjögren’s syndrome, and using HCQ for at least three years was associated with a reduced risk of developing DM [66] as a risk factor for PD [67]. However, in our study, patients with pSS receiving geniquin therapy had a higher risk of PD (1.608 times) and periodontal therapy (1.843 times) than those without pSS.

The potential role of other medications in treating PD in patients with pSS should be noted since numerous drugs are known to affect periodontal tissues [68]. Cholinergic parasympathomimetic agents (cevimeline and pilocarpine) can increase secretions by exocrine glands, including sweat glands, salivary glands, lacrimal glands, gastric glands, intestinal glands, and mucous cells in the respiratory tract [69]. In our study, patients with pSS had a higher rate of receiving cevimeline and pilocarpine but showed no significantly lower risk of PD in the Cox regression.

This study highlights a critical finding: pSS patients undergoing geniquin therapy are at the highest risk of subsequent PD diagnosis and periodontal therapy. This emphasizes the vital role of dental and medical professionals in identifying and managing periodontitis in pSS patients receiving geniquin therapy.

## Limitations

There are some notable limitations in this study. Firstly, PD and pSS diagnosis were solely reliant on ICD codes, lacking detailed information on PD (loss of attachment, personal health status, and medications with gingival hyperplasia side effects) and pSS (four of the six criteria for diagnosing pSS were not specified). Secondly, the NHIRD did not provide data on important socioeconomic factors and health-risk behaviors such as marital status, education, smoking, betel nut consumption, alcohol use, and other potential impacts of unmeasured factors (e.g., nutritional status, physical activity, family history, genetic, psychosocial, and detailed environmental factors). Lastly, due to statistical complexity, the study did not consider disease-modifying antirheumatic drug therapy or anti-inflammatory treatment.

## Conclusion

Patients diagnosed with pSS had an increased risk of developing subsequent PD and receiving periodontal treatment than patients without pSS, while pSS patients receiving geniquin therapy showed even higher risks. This result serves as a reminder to dentists and physicians that identification and treatment of PD in pSS patients receiving geniquin therapy is crucial. More comprehensive studies using a longitudinal approach are needed to better understand how autoimmune diseases could contribute to susceptibility to periodontitis and to characterize the interaction between host genetics and microbial diversity in the manifestations and progression of PD.

## Supporting information

S1 Table Sjögren’s syndrome with geniquin therapy vs Sjögren’s syndrome without geniquin therapy at the end of follow-up. (DOCX)
