# Supplementary material for: Association between geniquin therapy and the risk of developing periodontal disease in patients with primary Sjögren’s syndrome: A population-based cohort study from Taiwan
Source: PLoS One. 2024 Aug 7;19(8):e0305130. doi: 10.1371/journal.pone.0305130 (PMC11305530; doi:10.1371/journal.pone.0305130)
Supplement: S1 Table — (DOCX) [file pone.0305130.s001.docx]

S1 Table. Sjögren's syndrome with geniquin therapy vs Sjögren's syndrome without geniquin therapy at the end of follow-up.

| **pSS with geniquin therapy vs pSS without geniquin therapy at the end of follow-up** | | | | | | | |
| --- | --- | --- | --- | --- | --- | --- | --- |
| **Geniquin** | **Total** |  | **With** |  | **Without** |  | **P** |
| **Variables** | **n** | **%** | **n** | **%** | **n** | **%** |  |
| **Total** | 106,818 |  | 15,149 | 14.2 | 91,669 | 85.8 |  |
| **Periotontitis** |  | | | | | | <.001 |
| Without | 94,877 | 88.8 | 13,235 | 87.4 | 81,642 | 89.1 |  |
| With | 11,941 | 11.2 | 1,914 | 12.6 | 10,027 | 10.9 |  |
| **Periotontitis Treatment** |  | | | | | | <.001 |
| Without | 100,811 | 94.4 | 14,183 | 93.6 | 86,628 | 94.5 |  |
| With | 6,007 | 5.6 | 966 | 6.4 | 5,041 | 5.5 |  |
